# Personalized Circulating Tumor DNA Biomarkers Dynamically Predict Treatment Response and Survival In Gynecologic Cancers

**DOI:** 10.1371/journal.pone.0145754

**Published:** 2015-12-30

**Authors:** Elena Pereira, Olga Camacho-Vanegas, Sanya Anand, Robert Sebra, Sandra Catalina Camacho, Leopold Garnar-Wortzel, Navya Nair, Erin Moshier, Melissa Wooten, Andrew Uzilov, Rong Chen, Monica Prasad-Hayes, Konstantin Zakashansky, Ann Marie Beddoe, Eric Schadt, Peter Dottino, John A. Martignetti

**Affiliations:** 1 Department of Obstetrics, Gynecology and Reproductive Sciences, Icahn School of Medicine at Mount Sinai, New York, NY, United States of America; 2 Department of Genetics and Genomic Sciences, Icahn School of Medicine at Mount Sinai, New York, NY, United States of America; 3 Department of Oncological Sciences, Icahn School of Medicine at Mount Sinai, New York, NY, United States of America; 4 Department of Biostatistics, Icahn School of Medicine at Mount Sinai, New York, NY, United States of America; National Cancer Institute, UNITED STATES

## Abstract

**Background:**

High-grade serous ovarian and endometrial cancers are the most lethal female reproductive tract malignancies worldwide. In part, failure to treat these two aggressive cancers successfully centers on the fact that while the majority of patients are diagnosed based on current surveillance strategies as having a complete clinical response to their primary therapy, nearly half will develop disease recurrence within 18 months and the majority will die from disease recurrence within 5 years. Moreover, no currently used biomarkers or imaging studies can predict outcome following initial treatment. Circulating tumor DNA (ctDNA) represents a theoretically powerful biomarker for detecting otherwise occult disease. We therefore explored the use of personalized ctDNA markers as both a surveillance and prognostic biomarker in gynecologic cancers and compared this to current FDA-approved surveillance tools.

**Methods and Findings:**

Tumor and serum samples were collected at time of surgery and then throughout treatment course for 44 patients with gynecologic cancers, representing 22 ovarian cancer cases, 17 uterine cancer cases, one peritoneal, three fallopian tube, and one patient with synchronous fallopian tube and uterine cancer. Patient/tumor-specific mutations were identified using whole-exome and targeted gene sequencing and ctDNA levels quantified using droplet digital PCR. CtDNA was detected in 93.8% of patients for whom probes were designed and levels were highly correlated with CA-125 serum and computed tomography (CT) scanning results. In six patients, ctDNA detected the presence of cancer even when CT scanning was negative and, on average, had a predictive lead time of seven months over CT imaging. Most notably, undetectable levels of ctDNA at six months following initial treatment was associated with markedly improved progression free and overall survival.

**Conclusions:**

Detection of residual disease in gynecologic, and indeed all cancers, represents a diagnostic dilemma and a potential critical inflection point in precision medicine. This study suggests that the use of personalized ctDNA biomarkers in gynecologic cancers can identify the presence of residual tumor while also more dynamically predicting response to treatment relative to currently used serum and imaging studies. Of particular interest, ctDNA was an independent predictor of survival in patients with ovarian and endometrial cancers. Earlier recognition of disease persistence and/or recurrence and the ability to stratify into better and worse outcome groups through ctDNA surveillance may open the window for improved survival and quality and life in these cancers.

## Introduction

Gynecologic malignancies will be diagnosed in over 94,000 women in the U.S. and will result in close to 29,000 deaths this year alone [1]. The development of more sensitive and accurate biomarkers will be critical for both earlier detection of disease and more effective surveillance in the post-treatment setting. For example, in epithelial ovarian cancer, the most lethal female reproductive tract malignancy worldwide, most women will present with advanced stage disease which will be managed by surgical resection followed by combination platinum- and taxane-based chemotherapy. Misleadingly, using current detection technologies, 80% of these patients will appear to have a complete clinical response to primary therapy. In fact, more than half will develop disease recurrence within 18 months. Thus, a critical time period for initiating more aggressive treatment earlier and improving survival is potentially lost forever. The only currently available serum biomarker in gynecologic malignancies, CA-125, lacks the sensitivity and specificity for monitoring treatment response and early detection of recurrence [2,3]. Imaging modalities including computed tomography (CT) scanning are commonly used for disease surveillance but also lack sensitivity and often remain inconclusive or delayed in demonstrating progression of disease [4,5].

The absolute quantity of cell-free DNA (cfDNA) in patient serum as a marker of disease presence in gynecologic malignancies was first explored more than 10 years ago [6,7]. With the advent of new sequencing technologies and analytic techniques, the ability to detect circulating tumor-specific DNA (ctDNA), often referred to as the “liquid biopsy”, has evolved. The measurement of ctDNA has been shown to be an accurate reflection of disease presence and tumor evolution in several cancer types including breast, lung, colon, and stomach cancers [8–12]. Previous studies in gynecologic malignancies have primarily evaluated the presence of ctDNA at a single time point using pelvic washes, ascites, serum and plasma [13–15]. As a proof-of-principle study to demonstrate the ability to serially track disease, we recently used a tumor-specific fusion event to demonstrate the continued presence of ctDNA, and thus minimal residual disease, in a single patient over a four-year period in the face of repeatedly normal CA125 measurements [16]. Apart from this, longitudinal studies correlating ctDNA levels with tumor burden and clinical course in gynecologic cancers are lacking. Nonetheless, the theoretical dynamic, sensitive and specific nature of ctDNA as a biomarker suggests great potential for revolutionizing our approach to tumor surveillance in gynecologic cancers.

Here we describe a rapid and efficient pipeline that couples tumor-specific point mutation identification with droplet digital PCR-based ctDNA detection. We have tested this pipeline to detect and monitor tumor status in 44 women with gynecologic cancers. Specifically, using a combination of whole exome sequencing (WES) and directed gene sequencing panel, we have compiled tumor mutation profiles for a cohort of ovarian and endometrial cancer patients. Patient-derived panels of ctDNA biomarkers were generated and tested using droplet digital PCR, which we validated to be capable of detecting single copies of ctDNA per serum sample. The ctDNA results were compared against the current FDA-approved serum biomarker, CA125, CT scanning and the known surgical/clinical status of patients. This is the first study to demonstrate in gynecologic cancers that ctDNA can detect the presence of cancer earlier than other currently recommended testing modalities and that ctDNA levels at the completion of primary therapy are an independent predictor of both progression free (PFS) and overall survival (OS).

## Methods

### Patient Enrollment and Sample Collection

Forty-four patients with gynecologic, malignancies were enrolled and approved for participation after obtaining appropriate written informed consent as a part of our Institutional Review Board (IRB)-approved gynecologic cancer genomics program at the Icahn School of Medicine at Mount Sinai (Table 1). Tumor samples were collected at time of surgery. Blood samples were collected at time of surgery and again with each blood draw throughout the patient’s clinical course. Surgical tumor tissue was immediately flash frozen in liquid nitrogen after harvesting and serum was collected in BD Vacutainer SST II Advance Tubes (BD Diagnostics, Franklin Lakes, NJ). Within four hours after collection, blood samples were centrifuged for 10 minutes at 12,000g). Serum was transferred to a 15 ml Falcon tube and centrifuged at 1,200g for an additional 10 minutes to eliminate remaining cellular debris. Clinical data was extracted from patient medical records and included demographic variables, initial tumor site, histology, grade, stage, CA-125 levels and chemotherapeutic regimens. Data regarding surgical procedures, sites of metastatic disease, and tumor recurrences, were also collected. All patients with endometrial or ovarian cancer were eligible for enrollment if the necessary specimens according to the study protocol and relevant clinical data were available: CA-125 levels, computed tomography (CT) results, surgical outcomes and chemotherapy regimens.

**Table 1:** Baseline characteristics of all enrolled patients (N = 44).

Demographic criteria	Number of patients in each group	Percentage (%)
**Age at diagnosis (N = 44)**		
40–49 years	4	9.1
50–59 years	14	32.8
60–69 years	19	43.2
>70 years	7	15.9
**Race/Ethnicity (N = 44)**		
White, non-Jewish	22	50
Jewish	11	25
Black	5	11.4
Asian	2	4.5
Hispanic	1	2.3
Unknown/Other	3	6.8
**BRCA Status (N = 44)**		
Positive	11	25
Negative	8	18.2
Unknown	25	56.8
**Histology classification by primary site**		
*Ovary (N = 22)*		
Serous	21	95.5
Malignant Mixed Mesodermal Tumor	1	4.5
*Uterus (N = 17)*		
Papillary Serous	8	47.1
Malignant Mixed Mesodermal Tumor	3	17.6
Mixed	3	17.6
Clear Cell	2	11.8
Endometrioid	1	5.9
*Peritoneal (N = 1)*		
Serous	1	100
*Fallopian Tube (N = 3)*		
Serous	3	100
*Synchronous fallopian tube & uterine (N = 1)*		
	1	100
**Stage at diagnosis (N = 44)**		
1	5	11.4
2	2	4.5
3	23	52.3
4	13	29.5
Unstaged	1	2.3

### DNA Extraction Protocols

Genomic tumor DNA was extracted from approximately 25–50mg of tissue using the DNeasy Blood and Tissue Kit (Qiagen, GmbH, Hilden) according to the manufacturer’s instructions. Germline DNA was extracted from whole blood using the ArchivePure DNA Kit (5 Prime) according to the DNA purification protocol for whole blood, provided by the manufacturer. Quantification of genomic DNA was performed using by Qubit fluorometry. Circulating free (cfDNA) was extracted from 1mL of serum using the Circulating Nucleic Acid Kit (Qiagen, GmbH, Hilden) and eluted with 105 ul of DNase- and RNAse-free water.

### Identification of Somatic Mutations

Personalized tumor mutation profiles were identified for each patient’s tumor by either whole exome sequencing (WES) or a targeted gene sequencing approach. In both assays, genomic DNA (gDNA) from tumor tissue and a normal control were sequenced to identify genetic variants (SNVs and small indels) specific to the tumor only (somatic mutations). All sequencing was performed within the Department of Genetics and Genomics at the Icahn School of Medicine at Mount Sinai. Whole-genome libraries were prepared from gDNA using the NEBNext DNA Library Prep kit (New England Biolabs, Ipswich, MA), enriched to whole exome libraries using the SeqCap EZ Human Exome Library v3.0 capture system (Roche NimbleGen, Madison, WI), covering approximately 64 Mb of the genome where variants can be called; paired-end sequencing (2x100 nt reads) was done on Illumina HiSeq 2500 (Illumina, San Diego, CA) in either High Output or Rapid Run mode. Targeted gene sequencing was performed using Ion AmpliSeq^TM^ Cancer Hotspot Panel v2 (Life Technologies, Carlsbad, CA) using the Ion Torrent PGM sequencing technology to ~1000 to 3000X coverage. The Ion AmpliSeq^TM^ Cancer Hotspot Panel v2 interrogates 50 oncogenes and tumor suppressor genes across 207 amplicons (covering 21,820 nt where variants can be called). Based on experience, candidate somatic mutations were triaged for Taqman probe development if mutant allele fraction was ≥ 8% and passed manual review by inspecting raw read alignments in the IGV genome browser tool, then validated for final generation of probe by direct Sanger sequencing of tumor DNA and its paired peripheral blood mononuclear cell (PBMC) genomic DNA. Since WES data provided a larger number of candidate somatic mutations than was practical to validate, candidates with higher allelic fraction and better variant call quality were prioritized.

### PCR Assay Design and Validation

Custom TaqMan Assays were designed using the Life Technologies web-based design tool (www.lifetechnologies.com/order/custom-genomic-products). Assays contained unlabeled PCR primers (forward and reverse) in addition to VIC, TET or FAM labeled probes, which probed for the wild type and mutant variants respectively. Assays were then validated by quantitative PCR (qPCR) using TaqMan® Genotyping Master Mix (Life Technologies, Foster City, CA) on an ABI PRISM 7900 HT Sequence Detection System (Applied Biosystems, Foster City, CA). First, specificity was established by testing assays on tumor and matched PBMC genomic DNA. Linearity of each assay was then established by comparing each assay against a standard curve prepared from a series of serial dilutions of tumor DNA, ranging from 400–4000 genome equivalent copies specific to that assay.

### Mutation Quantification in Circulating Free DNA (cfDNA)

To further establish sensitivity, linearity and the lower limits of detection, developed assays were then tested by droplet digital PCR (RainDance Technologies, Billerica, MA) as per the manufacturer’s protocol. For this purpose, serial dilutions of tumor DNA, prepared for each assay, and ranging from 2–24 copies were spiked into a background of 100,000 copies of genomic wild type DNA. Once the lower limit of detection was established for each assay, ddPCR was performed on cfDNA extracted from patient serum and/or plasma. Twenty μl of eluted cfDNA was used for each PCR reaction, representing 200 μl of serum. The total PCR reaction volume was 50 μl (generating 10^6 droplets). PCR conditions were as follows: 95C for 10 minutes for one cycle; 95C (15 seconds) and 58C (one minute) with a step for 45 cycles each; and 98C (10 minutes). The PCR product was then introduced into the RainDrop Sense instrument for quantification of wild type and mutant copy numbers. Each cfDNA sample was analyzed by at least two replicates. To establish assay reproducibility and the efficiency of cfDNA extraction, we spiked each patient blood sample with a known concentration of Lambda DNA digested with HindIII phage (Qiagen, GmbH, Hilden) prior to cfDNA isolation. qPCR was used to quantify the 125 and 535 bp DNA fragments of the λ- HindIII. Based on λ DNA extraction efficiency, the recovery efficiency for cfDNA extractions used in these studies was between 60 and 90%.

### Statistical Analysis

To assess the ability of ctDNA and CA125 to predict the presence of tumor on CT imaging and at time of surgery, the parameters of sensitivity and specificity as well as corresponding 95% confidence intervals were estimated using log-binomial models that took into account the correlations that arose from there being multiple measurements on the same patient over time. Predicted probabilities from these fitted models were then used in logistic regression models to estimate and compare the areas under the Receiver Operating Characteristic (ROC) curves (AUCs) for both ctDNA and CA125. Overall survival (OS) and progression free survival (PFS) were estimated using the method of Kaplan-Meier and compared with the log-rank test, between patients with levels of ctDNA that were either present or absent following surgical resection and adjuvant therapy. All statistical analyses were performed using SAS version 9.3 (SAS Institute, Inc., Cary, NC). Hypothesis testing was two-sided and conducted at the 5% level of significance.

## Results

### Clinicopathologic Characteristics of Patients and Their Tumors

Forty-four patients undergoing treatment for gynecologic cancer were enrolled for this study and their cancer type and clinicopathologic information is shown in Table 1. Tumor-derived DNA, germline DNA, ctDNA and clinical data were available for all patients. All ovarian tumors were high-grade serous histology with the exception of one malignant mixed mesodermal tumor. Although the majority of endometrial cancers were high grade, the histologic subtypes were varied. For the purposes of our analysis high-grade serous tumors of the fallopian tube, peritoneum and ovary were grouped together as pelvic high-grade serous carcinomas (HGSC). The majority of tumors sequenced were collected at time of primary surgery (n = 40) while four tumor samples were from recurrences.

### Identification of Somatic Genomic Variants and Development of Personalized ddPCR Assays

**Fig 1:**
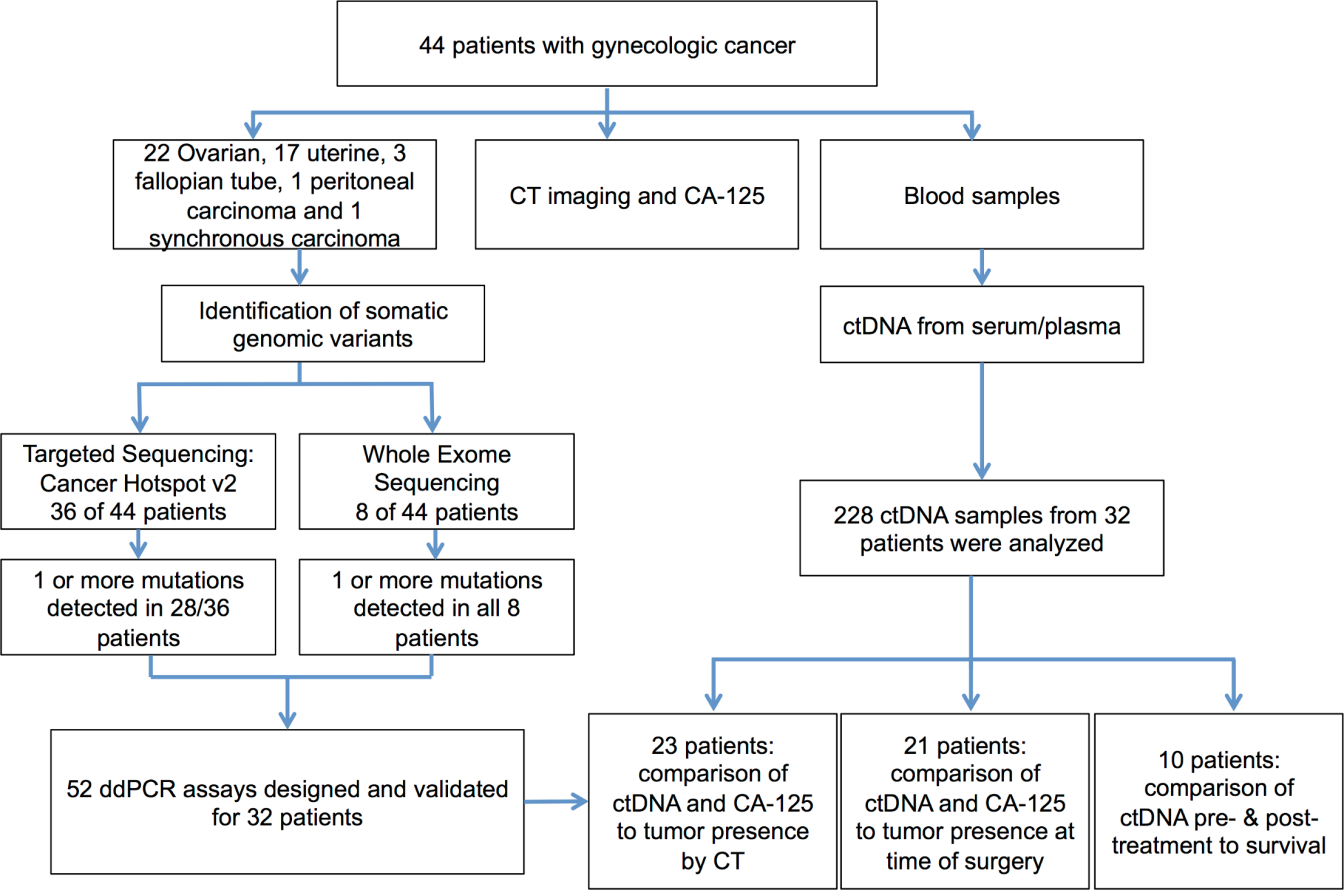
Overview of patient enrollment, clinical follow-up and personalized ctDNA analysis.

An overview of our personalized ctDNA analysis pipeline is shown in S1 Fig. Using a combination of WES and targeted panel sequencing, we first aimed to identify significant tumor-specific mutations for developing droplet digital PCR (ddPCR)-based assays. Eight tumors were sequenced using WES data from Illumina paired-end sequencing collected on the HiSeq 2500 and 36 were sequenced using the targeted Ion AmpliSeq™ Cancer Hotspot Panel v2 using the Ion Torrent PGM sequencer. All tumors sequenced using WES were ovarian tumors. Serum samples were available from either primary or secondary surgery for all patients. Longitudinal serums were available for 23 patients. ctDNA was extracted from a total of 228 individual time points with an average of 7.8 time points per patient (Fig 1).

Candidate mutations were identified for 36 patients (S1 Table). Of the mutations discovered using the targeted panel, the most commonly mutated gene was TP53 (23/36 patients; 66%), which always occurred in high-grade serous histology of both ovarian and endometrial tumors. Mutations were also identified, albeit at lower frequencies, in PTEN, PIK3CA, MET, KRAS, FBXW7 and BRAF. Multiple candidate mutations were identified for 16 patients. All candidate mutations were confirmed by Sanger sequencing of the respective tumors.

In total, 52 unique assays, out of 55 designed, were validated for sensitivity, specificity and linearity using quantitative PCR (qPCR), representing 32 patients for which ctDNA quantitative studies could be performed. The robustness of the different assays generated for each of the patients was demonstrated by a number of different metrics. For all 52 assays, the correlation coefficient for linearity, R^2^, was greater than 0.99 (mean, 0.9988, S2A Fig). The lower limits of detection were established for each personalized assay using spiked tumor DNA from the respective patient with known mutations and tested using 2–24 mutant copies in a background of 100,000 wild-type copies of their germline DNA. Based on these tests, sensitivity to discriminate between wild type and mutant sequence for all probes used in this study ranged between 0.01% and 0.002%. Quantification of specific tumor mutant copies in cfDNA was highly correlated in ddPCR replicates (Pearson r: 0.9938, S2C Fig). There was also a high linear correlation between allelic fraction as determined by ddPCR and targeted sequencing (Pearson r: 0.95) and this correlation was statistically significant p = 4.47E-15 (S2D Fig).

### Estimating the Sensitivity and Specificity of ctDNA to Predict Tumor Presence

We then evaluated the ability of serially-collected ctDNA to screen for clinical presence of tumor. Concurrently, we also compared the currently used FDA-approved test, CA-125. To establish a relative ground truth for tumor presence, we used CT imaging and findings at the time of surgery. While relatively imprecise, this did allow us to identify a number of important findings. Data comparing ctDNA and CA-125 levels to the presence of tumor on CT imaging was available across 53 time points for 23 patients.

**Fig 2:**
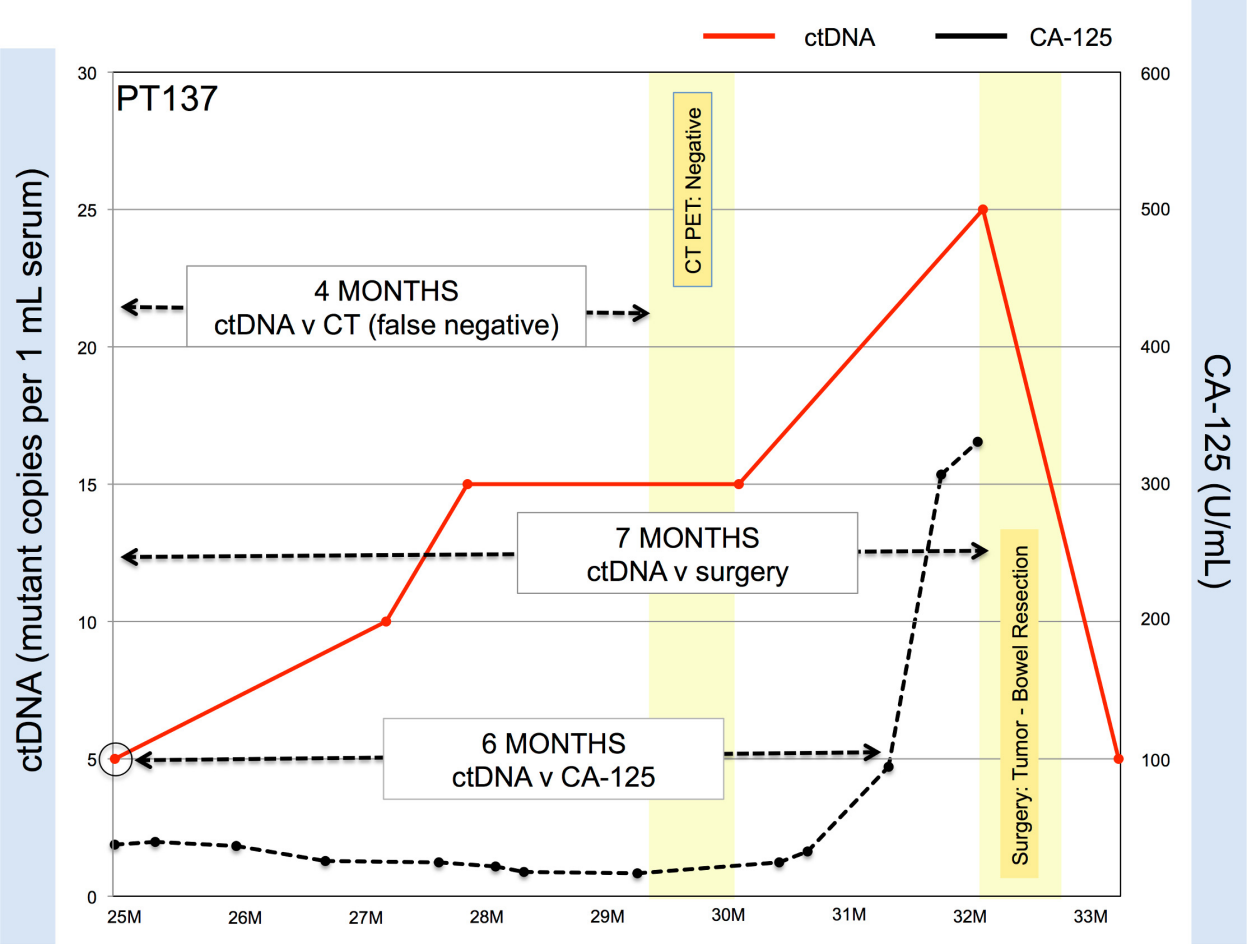
Circulating tumor DNA can detect relapse earlier than CA125 and CT scan imaging. In this representative example, increases in ctDNA levels in Patient 137 levels precede a rise in CA-125 levels by six months and pre-date positive identification of tumor growth requiring bowel resection seven months later. CT scanning was non-specific and patient was brought to the operating room for exploratory surgery, which revealed the presence of tumor.

The sensitivity and specificity of ctDNA to predict the presence of tumor on the paired CT scan was 0.91 [0.73–0.97] and 0.60 [0.31–0.83], respectively (Table 2). Similar results were obtained for CA-125 and CT scanning (Table 2). The low specificity of ctDNA was surprising to us and so we examined this in greater depth. The discrepancy was found to be the result of a lack of concordance between tests in six patients. Specifically, all six patients had detectable levels of ctDNA but negative CT imaging results within two weeks of the ctDNA blood draw. Interrogation of their complete history revealed that all six patients were later found to have surgery proven tumors that had not been detected by CT scanning. Moreover, analysis of patient data revealed that on average, ctDNA predicted recurrence approximately 7 months (range: 1–11 months) earlier than CT imaging (Fig 2).

**Table 2:** Levels of ctDNA correlate with tumor presence and are as sensitive and specific as CA-125.

Detection modality	Screening Marker	Sensitivity 95% CI	Specificity 95% CI	AUC 95% CI
CT	ctDNA	0.91 [0.73–0.97]	0.60 * [0.31–0.83]	0.76 [0.62–0.90]
CT	CA125	0.85 [0.61–0.95]	0.62 * [0.37–0.82]	0.73 [0.57–0.88]
Surgery	ctDNA	0.81 [0.60–0.92]	0.99 [0.81–0.99]	0.80 [0.59–1.00]
Surgery	CA125	0.82 [0.62–0.93]	0.63 [0.17–0.93]	0.74 [0.41–1.00]

Sensitivity and specificity of ctDNA and CA-125 were calculated at 95% CI and compared against CT imaging results and presence or absence of detectably tumor at time of surgery.

*Six patients were found to have detectable ctDNA in the face of negative or non-specific CT scans. CI: confidence interval. AUC: area under the curve.

CA-125 and ctDNA levels were then compared to the presence of tumor at time of surgery across 30 time points for 21 patients. When compared to the presence of tumor at surgery, ctDNA had a sensitivity of 0.81 [0.60–0.92] and a specificity of 0.99 [0.81–0.99]. By comparison, the sensitivity and specificity of CA-125 was 0.82 [0.61–0.93] and 0.64 [0.17–0.94], respectively. Areas under the ROC curves were calculated for ctDNA and CA-125 and were 0.91 [0.83–0.99] and 0.70 [0.44–0.95], respectively. Although these results suggest that ctDNA could be a more accurate diagnostic test than CA-125, the difference in ROC curves was not statistically significant (p = 0.3575) [Table 2].

### ctDNA Levels following Initial Treatment Are Predictive of Survival Differences

**Fig 3:**
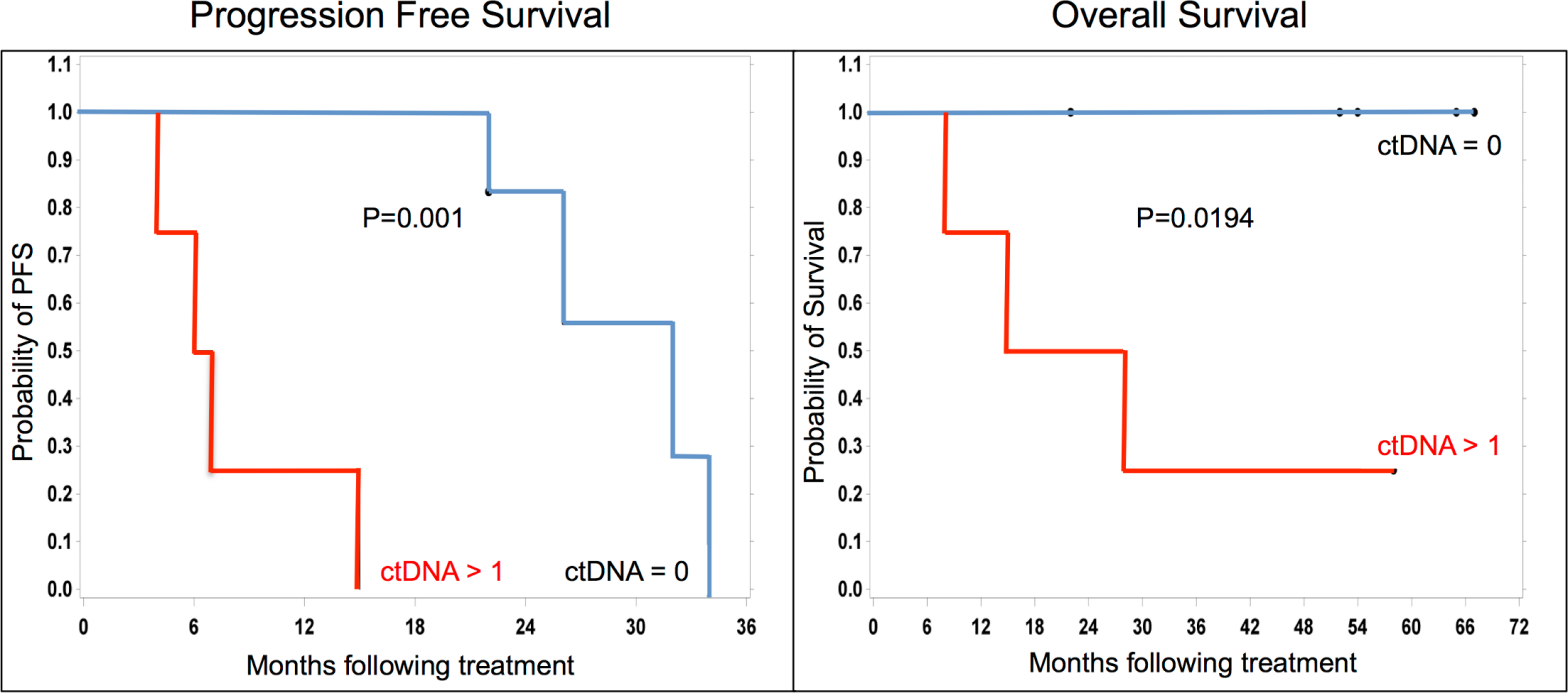
Undetectable levels of ctDNA following initial treatment are associated with improved survival. Kaplan–Meier analysis of progression-free (left panel) and overall survival (right panel) between individuals with undetectable (ctDNA = 0; blue lines) and detectable ctDNA (≥ 1; red lines). Significant differences in progression-free survival (p = 0.001) and overall survival (p = 0.0194) between undetectable and detectable groups.

We next assessed whether ctDNA levels following surgical resection and adjuvant therapy had prognostic significance. Pre- and post-treatment ctDNA levels were analyzed for 10 patients and compared to PFS and OS. Current clinical status, dead of disease (DOD), alive with disease (AWD) and no evidence of disease (NED), was available for all 10 patients (Table 3). Undetectable levels of ctDNA following initial adjuvant treatment were associated with both improved PFS (p = 0.0011 Kaplan Meier) and OS (p = 0.0194; Fig 3). The median PFS difference was just over 2 years (six months versus 32 months). All four patients with average post-treatment ctDNA levels ≥ 10 copies/ml are DOD, whereas the one patient with ctDNA = 5 copies/ml, who also intriguingly had very low levels of ctDNA pre-treatment, is AWD. Of the five patients with undetectable ctDNA levels post-treatment, none have died of their disease, three are AWD and two are NED. Two of the patients have already survived beyond 5 years. We noted no survival differences based on ctDNA pre-treatment values.

**Table 3:** Serial measurement of pre- and post-treatment ctDNA for predicting survival.

Patient	Primary site	PFS (months)	OS (months)	Current Survival Status	ctDNA Pre-Treatment [avg]	ctDNA Post-Treatment [avg]
PT217	Ovary	0	15	DOD	3345	30
PT208	Ovary	15	28	DOD	10	10
PT186	Uterus	0	29	DOD	60	15
PT247	Uterus	0	8	DOD	195	15
PT137	Ovary	0	57	AWD	1	5
PT158	Ovary	23	52	AWD	77	0
PT105	Ovary	32	64	AWD	385	0
PT078	Ovary	33	65	AWD	0	0
PT229	Ovary	22	22	NED	0	0
PT230	Ovary	22	22	NED	40	0

Pre-treatment serums were drawn at the time of surgery and prior to initiation of chemotherapy. Post-treatment serums were drawn after the completion of the final cycle of adjuvant chemotherapy. All copy number values represent the average of at least two measurements. DOD: died of disease. AWD: alive with disease. NED: no evidence of disease

## Discussion

We demonstrate that serial measurement of ctDNA is a surveillance biomarker in gynecologic cancers that is as sensitive and specific as the FDA-approved serum biomarker CA-125 and can detect disease relapse months earlier than CT scanning. Moreover, the measurement of ctDNA levels at the time of completion of initial therapy, debulking surgery and combination platinum/taxane doublet chemotherapy, provides a novel predictor of survival differences in patients. Specifically, undetectable levels of ctDNA were associated with both improved progression free and overall survival. In our study population, the difference in PFS was just over two years between women with detectable and undetectable levels of ctDNA. These differences, earlier detection than CT imaging and separation of women with worse and better survival, is striking since the theoretical basis for cancer surveillance is that earlier detection results in better therapeutic options and outcomes. What is currently known is that at present, and relying on clinical examination, CA-125 measurements and imaging, the majority of ovarian cancer patients following initial treatment are diagnosed as having a complete clinical response. Despite this, more than half of these women will develop disease recurrence within 18 months.

CA-125 and CT imaging are the most frequently used surveillance modalities in ovarian and endometrial cancers. Even so, CA-125 is considered optional and CT imaging is recommended only as indicated clinically by the National Comprehensive Cancer Network (NCCN) guidelines [17]. Upwards of 50% of patients with ovarian cancer with normal CA125 levels following chemotherapy nonetheless have persistent disease but distinguishing between patients solely on this biomarker is currently not possible [18]. For the ~50% of patients with persistent disease, the increased sensitivity and specificity afforded by a genomics-based approach might allow additional sub-categorization of patients to help differentiate disease and therapeutic subtypes. CA-125 is also widely expressed in other tissues and is elevated in benign processes such as endometriosis, uterine leiomyomata, benign adnexal masses, tubal inflammation, liver disease, heart failure, menses, and pregnancy, limiting its utility in surveillance of ovarian cancer [19]. Finally, with a half-life estimated to range from 9–44 days [20], CA-125 cannot offer as rapid a response to tumor volume changes as ctDNA. On the other hand, while CT imaging is the standard approach in the evaluation of recurrence of disease, in our cohort there were six instances of negative CT imaging with detectable ctDNA in the face of residual disease. Moreover, and as noted above, a rise in ctDNA preceded radiologic evidence of tumor recurrence, on average by 7 months.

In a number of other cancer types, ctDNA levels have been demonstrated to more closely correlate with changes in tumor volume than currently available biomarkers [8], however longitudinal human studies correlating ctDNA levels with tumor burden in gynecologic malignancies had been lacking. Previously, our group reported on the detection of genetic rearrangements, specifically fusion events, and their use as ctDNA assays to retrospectively monitor a patient with advanced stage ovarian cancer [16]. The rationale for initiating our study was that despite this patient’s history of seeming clinical remission based on multiple negative clinical, biochemical and radiologic studies she had multiple recurrences. For a four-year period, the patient underwent primary debulking surgery and chemotherapy, tumor recurrences, and multiple chemotherapeutic regimens. Blood samples were longitudinally collected and stored in our Biobank as part of a research program. Whereas postsurgical CA125 levels were elevated only three times for 28 measurements, the specific fusion ctDNA biomarker was readily detectable in all of these same blood samples and in the tumor recurrences. Although we did not directly correlate ctDNA quantity with tumor burden, the tumor-specific fusion ctDNA continued to be detectable at times of apparent clinical remission, demonstrating the ability for ctDNA to detect occult disease in ovarian cancer.

In this present study, we now demonstrate the ability to sensitively detect single point mutations by digital PCR at specificities even beyond 0.01% from a complex sample. Using this pipeline, once the patient-specific ctDNA probes have been generated and validated, testing of serum samples can be completed within hours and is easily incorporated into a surveillance program (**S3 Fig**). Our approach does require prior knowledge of tumor-specific genetic alterations in each patient’s tumor. In this study, we used a combination of WES and a targeted panel which targets 50 commonly mutated oncogenes and tumor suppressors. This combined approach allowed us to generate ctDNA probes for 82% of the women in our study. As expected, WES identified mutations in 8/8 of the tumor samples. The cancer panel that we used allowed us to interrogate 207 amplicons across 50 oncogenes and tumor suppressor genes at a very high coverage and resulted in the identification of mutations in 28/36 (78%) samples. In the future, the use of sequencing panels specifically focused on ovarian and endometrial cancers should allow for an even more robust and efficient identification platform.

The goal of these next-generation surveillance studies is to detect disease recurrence earlier so that patients will be more optimal surgical candidates, their treatments more effectively personalized and, if and when necessary, triaged for therapeutic trials. Conversely, for some women, the use of ctDNA-based biomarkers could reduce the number of unnecessary interventions, result in less over treatment and circumvent the financial cost and radiation associated with CT imaging studies. Ultimately, now that ctDNA has been shown to detect residual disease earlier and can distinguish between women with worse and improved survival in ovarian and endometrial cancers, clinical studies defining the utility of ctDNA as a surveillance tool in gynecologic cancers can now be designed.

## Supporting Information

S1 Fig Overview of the ctDNA analysis pipeline. (TIF)

S2 Fig Accuracy and reproducibility of ddPCR assay in PT208 (TP53 mutation; Chr17: 7577539; G>A) for ctDNA detection and quantification. (A) Assay linearity was determined by analyzing serial fold tumor allele fraction dilutions. Mutant copies detected by the ddPCR system were supported by an R^2^ of 0.99. (B) The lower limit of detection for this assay was established by spiking serial tumor DNA dilutions in a base of 100000 copies of genome equivalents from the patient's germline DNA. Fractional mutation concentration percentages ranged from 0.025–0.002 and were analyzed by ddPCR. Mutation fraction was detected as low as 0.002%. (C) Replicate reproducibility for ctDNA detection. Pearson correlation between individual replicates is calculated and shown (Pearson r = 0.99) (D) Agreement of mutant allelic fraction determination between sequencing and ddPCR (p = 4.47E-15).
(TIF)

S3 Fig Current and proposed gynecologic surveillance algorithm. (TIF)

S1 Table Candidate tumor specific mutations identified by WES and targeted sequencing. Samples interrogated by WES are highlighted with an asterisk. All mutations shown were confirmed by Sanger sequencing of the relevant tumor sample.
(XLSX)
