# Supplementary figures and images for: Personalized Circulating Tumor DNA Biomarkers Dynamically Predict Treatment Response and Survival In Gynecologic Cancers
Source: PLoS One. 2015 Dec 30;10(12):e0145754. doi: 10.1371/journal.pone.0145754 (PMC4696808; doi:10.1371/journal.pone.0145754)

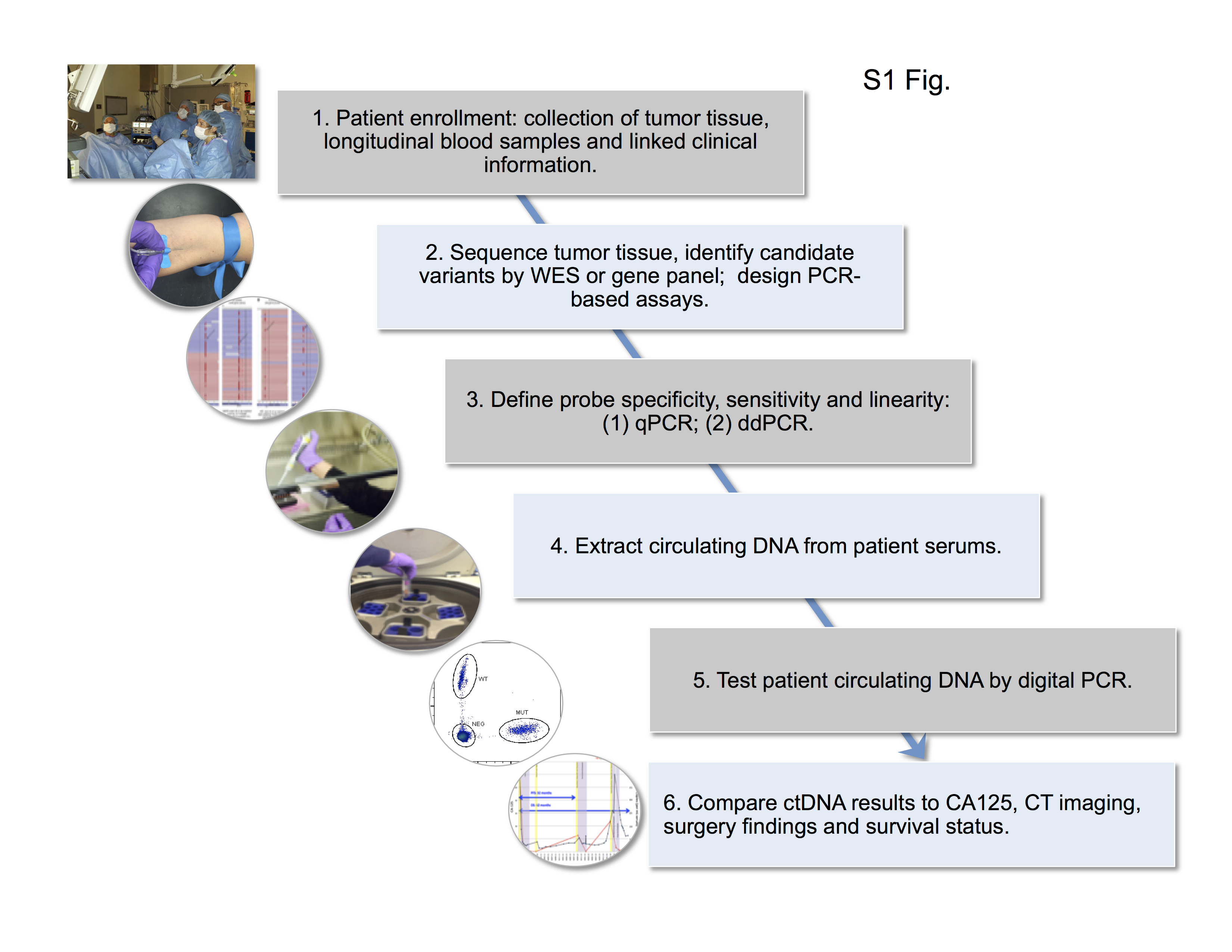

Supplement: S1 Fig — (TIF) [file pone.0145754.s001.tif]

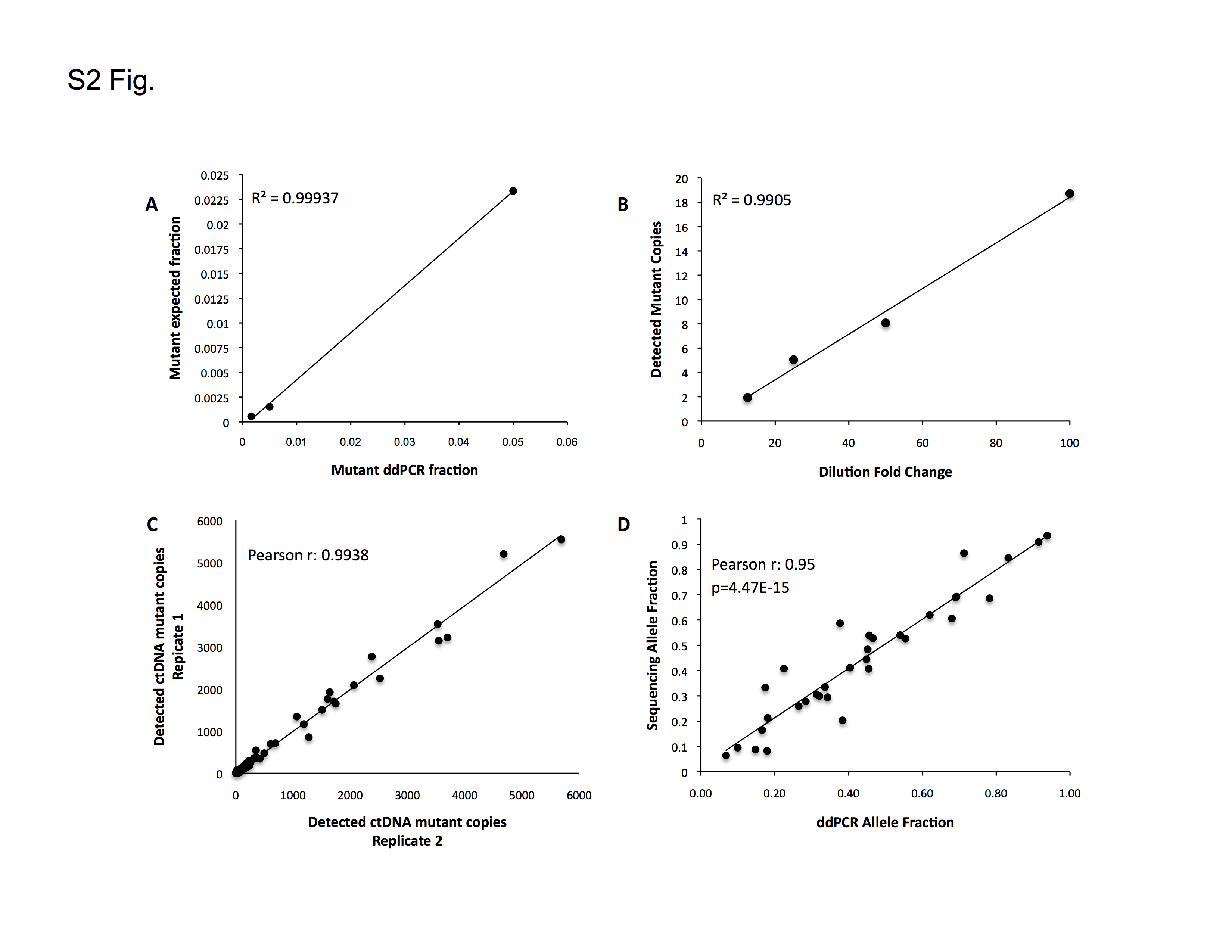

Supplement: S2 Fig — (A) Assay linearity was determined by analyzing serial fold tumor allele fraction dilutions. Mutant copies detected by the ddPCR system were supported by an R2 of 0.99. (B) The lower limit of detection for this assay was established by spiking serial tumor DNA dilutions in a base of 100000 copies of genome equivalents from the patient's germline DNA. Fractional mutation concentration percentages ranged from 0.025–0.002 and were analyzed by ddPCR. Mutation fraction was detected as low as 0.002%. (C) Replicate reproducibility for ctDNA detection. Pearson correlation between individual replicates is calculated and shown (Pearson r = 0.99) (D) Agreement of mutant allelic fraction determination between sequencing and ddPCR (p = 4.47E-15). (TIF) [file pone.0145754.s002.tif]

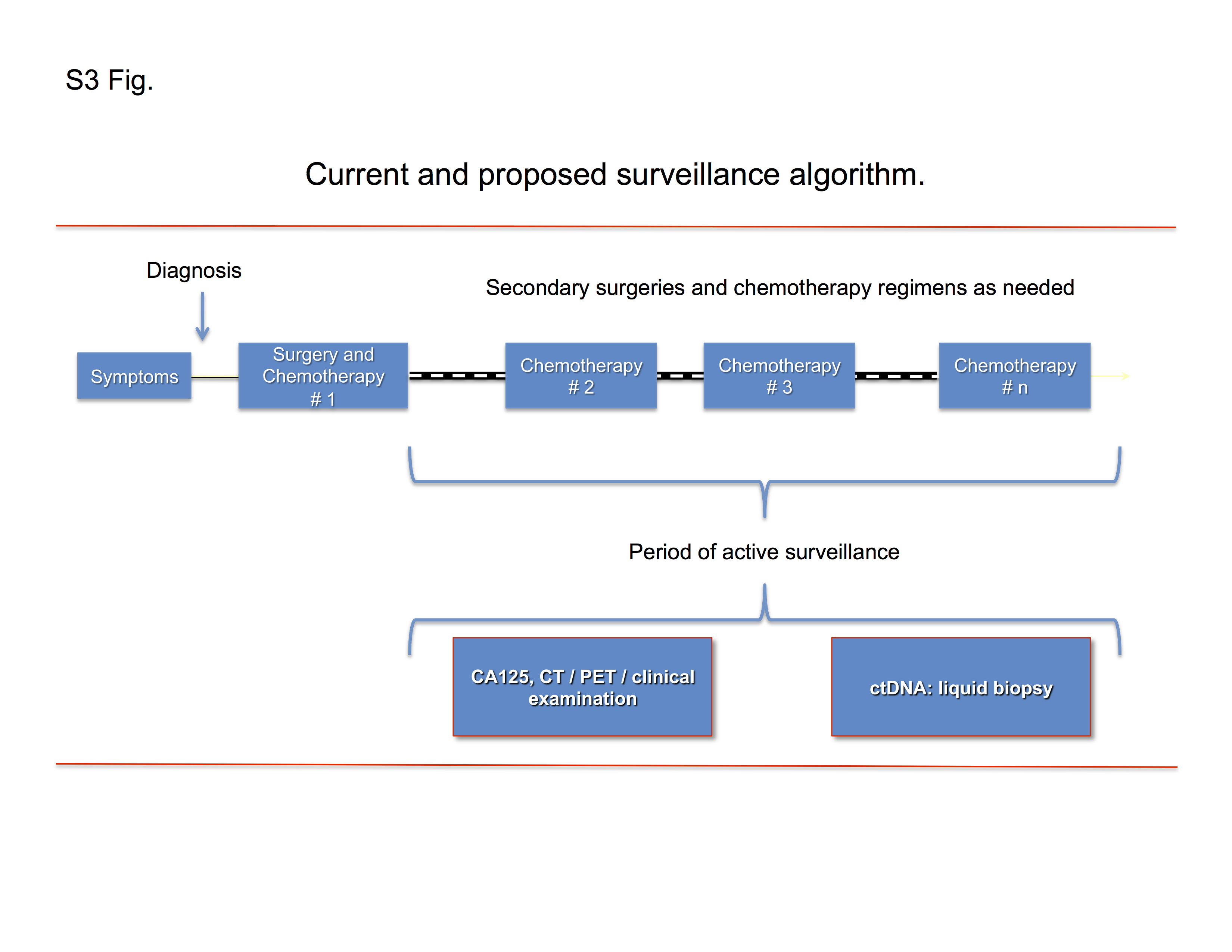

Supplement: S3 Fig — (TIF) [file pone.0145754.s003.tif]
